# Multiple Primary Malignant Neoplasms in African Americans: A Case Series and Literature Review

**DOI:** 10.7759/cureus.21585

**Published:** 2022-01-25

**Authors:** Fady Sidhom, Devon Jackson, Ahmed Ali, Babak Shokrani, Lekidelu Taddesse-Heath

**Affiliations:** 1 Internal Medicine, Howard University Hospital, Washington, DC, USA; 2 Pathology, Howard University Hospital, Washington, DC, USA; 3 Medical Oncology, Howard University Hospital, Washington, DC, USA

**Keywords:** non-small-cell lung carcinoma (nsclc), colorectal cancer, african americans, diagnosis of multiple myeloma, hepatocellular carcinoma (hcc), multiple primary cancer, multiple primary neoplasms, multiple primary malignant neoplasm

## Abstract

Multiple primary malignant neoplasms (MPMNs) or multiple primary malignancies are defined as two or more histologically distinct malignancies present in the same individual. While second or higher-order malignancies account for approximately 18% of all cancers in the United States, it is reasonable to presume that MPMNs are now occurring more frequently than previously reported. Underserved groups such as blacks and Hispanics may represent a high proportion of these underreported cases due to well-established health disparities. Although the role of health disparities has been well established in single primary malignancies, less is known on racial differences in patients with multiple primaries. In comparing MPMNs by race, blacks have lower survival rates compared to white patients. Moreover, despite the lower overall incidence of MPMNs in blacks compared to white patients, when broken down by the specific types of cancers and gender, there are significant racial disparities in the incidence of prostate cancer and possibly other cancers. Further research and case reports are required to explore the risk factors of developing MPMNs in these groups. Our case series explores three African American patients with MPMNs that are rarely described in the literature and outlines the management challenges of treating multiple malignancies.

## Introduction

While multiple primary malignant neoplasms (MPMNs) were first described in 1889 [1], their incidence in recent years has increased due to improvements in screening and therapies leading to longer life expectancy in cancer patients. Although cancer survivors are known to have a higher risk for developing cancer, it is unknown if this risk is the same across all ethnicities. As the incidence of MPMNs increases, special focus should be placed on underserved groups at risk for developing multiple malignancies. While blacks and Hispanics are known to have lower five-year overall survival rates [2], less is known about the differences in risk for these groups as it pertains to developing a second primary malignancy. Overall, in single primary cancers, blacks are known to have higher incidence and lower survival rates, but it is not well studied if this carries over into multiple primary malignancies as well. Several factors play into the development of multiple primary malignancies, with racial disparities possibly playing an important role.

In a large cohort of African Americans with multiple myeloma, it was found that blacks had a lower overall incidence of second primary malignancies than white patients, but there was a higher incidence of prostate cancer in black males [3]. This finding suggests that when broken down by gender and specific types of cancers, we may find more racial disparities in MPMNs.

This case series explores the rare presentations of MPMNs in three African American patients (Case A was previously published [4]) and the possible impact of race on developing MPMNs.

## Case presentation

Case A

**Figure 1:**
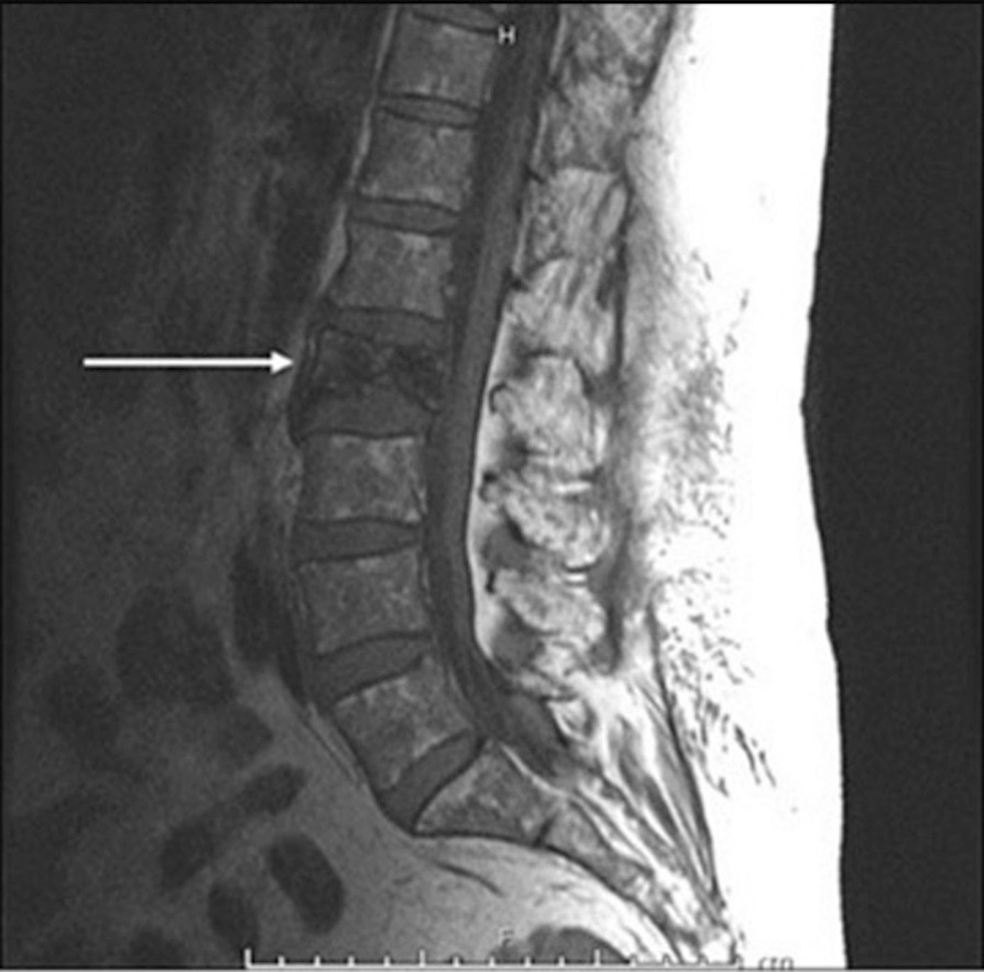
MRI of the lumbar spine with contrast showing L2 increased heterogeneity (white arrow), suggestive of metastasis. MRI: magnetic resonance imaging

**Figure 2:**
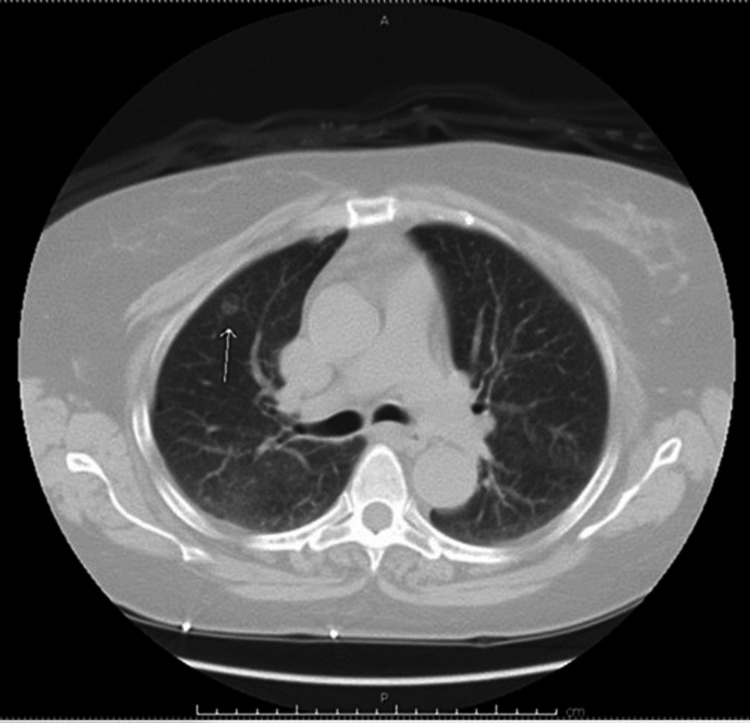
CT of the chest without contrast showing right upper lobe nodule (white arrow). CT: computed tomography

**Figure 3:**
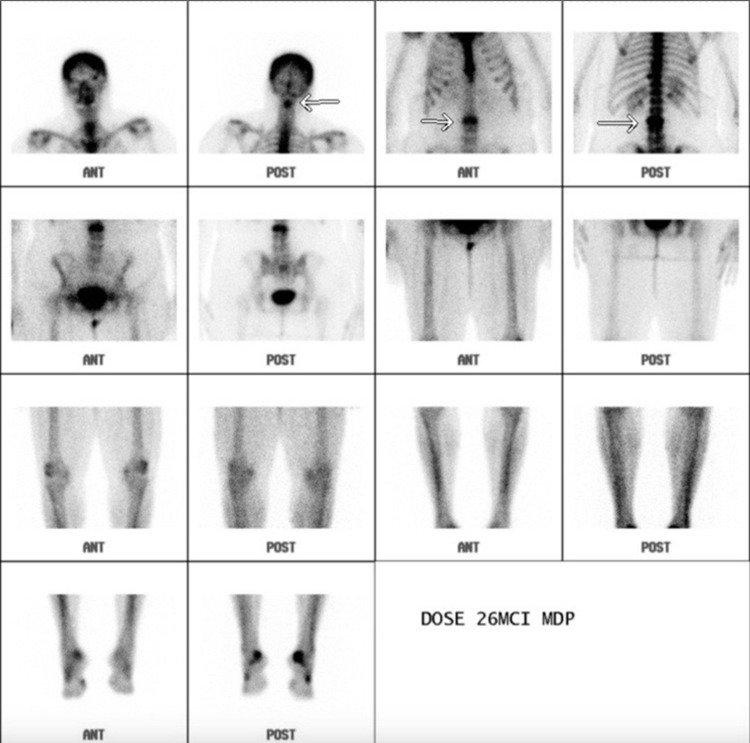
Bone scan showing solitary metastasis in the L3 vertebral body.

**Figure 4:**
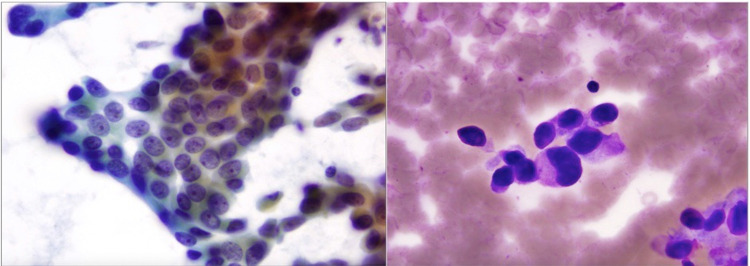
Lumbar vertebrae and lung nodule biopsies. Left image: fine-needle aspiration of the lumbar spine showing syncytial groupings and single-lying tumor cells with occasional nuclear crowding and overlapping. Right image: fine-needle aspiration of the right upper lobe lung showing tumor cells with high nuclear-cytoplasmic ratio and enlarged hyperchromatic and pleomorphic nuclei.

**Figure 5:**
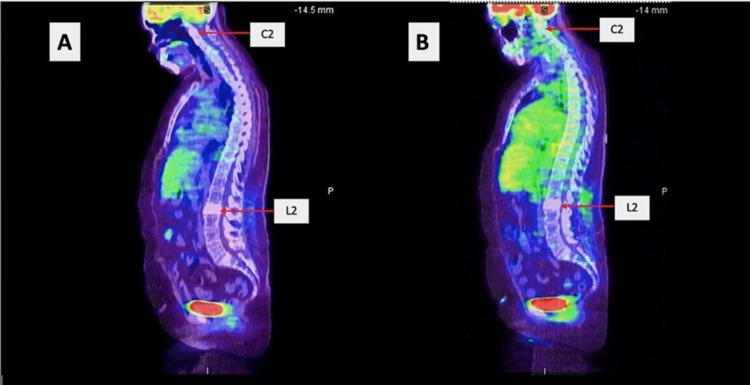
(A) Initial PET scan showing diffuse sclerotic lesion in the C2 and L2 vertebrae and no other hypermetabolic areas. (B) Follow-up PET scan showing stable sclerotic lesions and no hypermetabolic activity. PET: positron emission tomography

**Figure 6:**
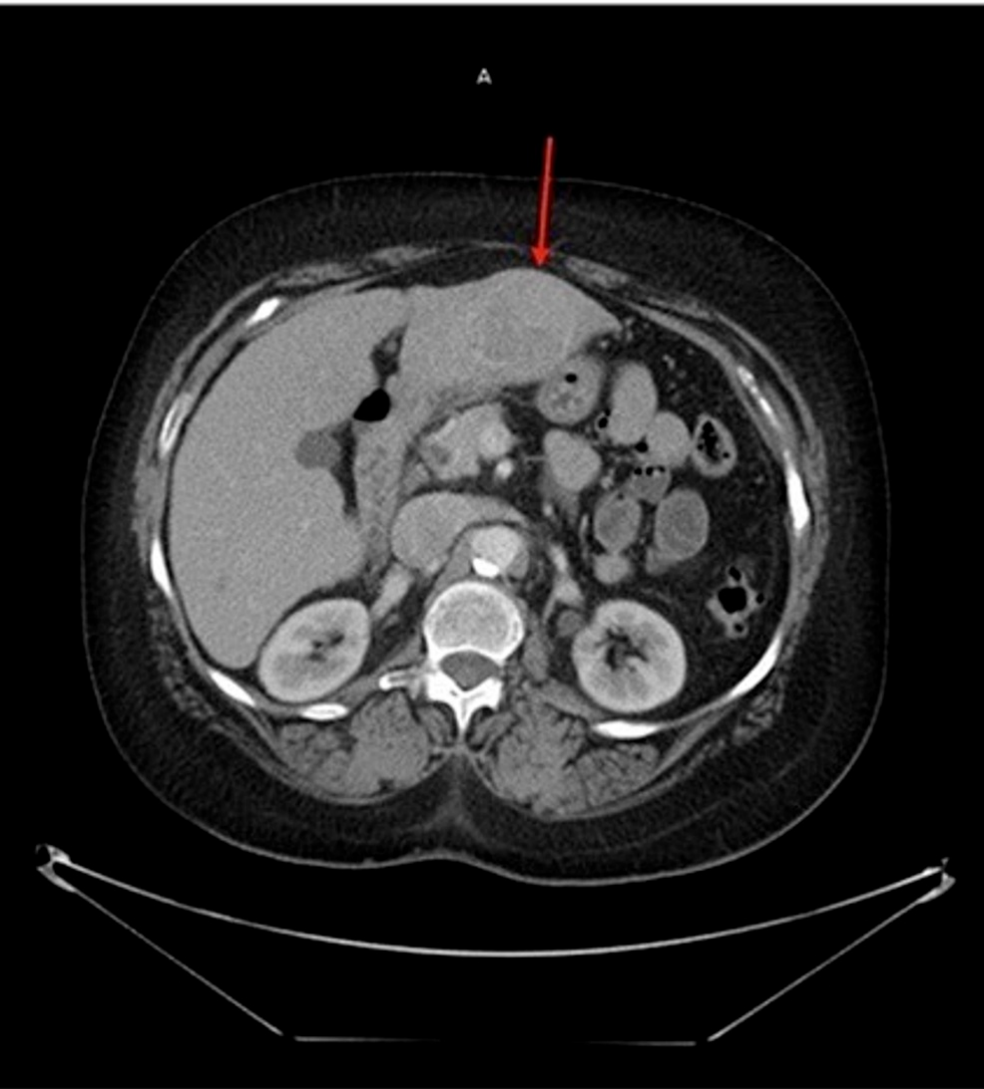
CT of the chest without contrast showing left hepatic lobe lesion with heterogeneous enhancement (red arrow). CT: computed tomography

**Figure 7:**
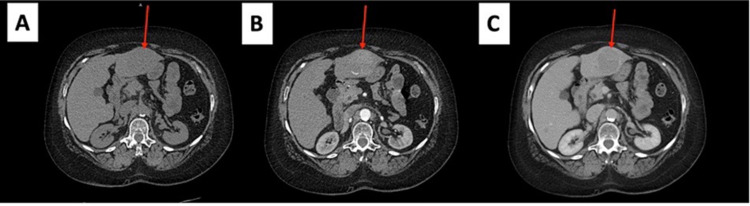
Triple-phase CT scan of the liver. A. Non-contrast image showing an isodense mass (arrow) in the left lobe of the liver. B. Arterial phase image showing a hypodense/heterogeneous mass. C. Fast washout of the tumor mass in the portal venous phase. CT: computed tomography

A 69-year-old African American female with a history of hepatitis C and cirrhosis initially presented with a complaint of worsening back pain for one year. An X-ray of the lumbar spine showed vertebral body sclerosis. Magnetic resonance imaging (MRI) was done for further evaluation and showed increased heterogeneity in the L2 vertebrae with a decrease in signal intensity compared to the remaining vertebrae, suggestive of metastasis (Figure 1). Computed tomography (CT) of the chest was also obtained and showed two nodules in both lungs measuring 1 cm in the right lung and 1.8 cm in the left lung (Figure 2). A subsequent bone scan showed solitary metastasis in the L3 vertebral body (Figure 3). A biopsy of the right upper nodule was positive for adenocarcinoma (Figure 4), and an L2 vertebral biopsy showed metastatic adenocarcinoma consistent with primary lung cancer (Figure 4). Radiation therapy to the L1-L3 spine was commenced using three-dimensional conformal radiotherapy. Immunostaining showed thyroid transcription factor-1 (TTF1) expression confirming primary lung adenocarcinoma, as well as an epidermal growth factor receptor (EGFR) mutation. Based on the genetic testing, the patient was started on gefitinib and denosumab. To assess for progression and response to treatment, a positron emission tomography (PET) scan was done which showed sclerotic lesions in L2 and C2 vertebral bodies (Figure 5). A follow-up PET scan after one year showed no hypermetabolic activity with stable sclerotic lesions (Figure 5). A surveillance CT of the chest, abdomen, and pelvis performed a year later was significant for a new liver mass, suggestive of a possible hemangioma (Figure 6). A subsequent triple-phase CT of the abdomen confirmed a liver mass with enhancement (Figure 7). A liver biopsy was performed which confirmed the diagnosis of hepatocellular carcinoma (HCC). Alpha-fetoprotein (AFP) and carcinoembryonic antigen (CEA) tumor markers were found to be elevated. Next-generation sequencing (NGS) showed a gatekeeper mutation of the T790M missense variant (Exon 20), and the patient was subsequently switched to osimertinib. The patient was also evaluated for surgical interventions but was deemed a non-surgical candidate due to her history of hepatitis C and cirrhosis. The patient underwent a transarterial radioembolization (TARE) treatment of her HCC with a good response.

Case B

**Figure 8:**
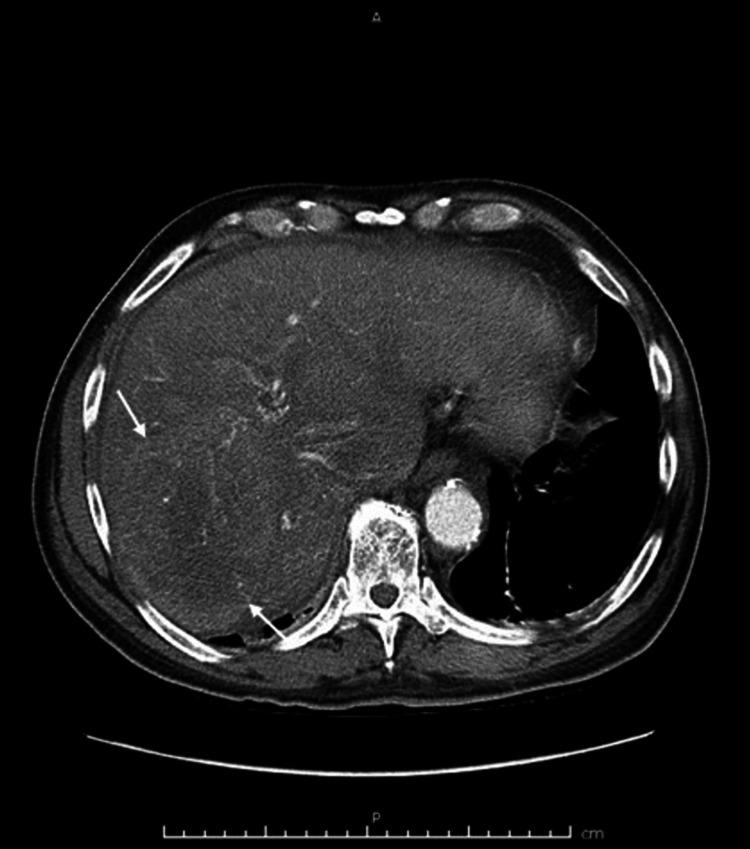
CT angiogram of the abdomen showing lesion in the right liver lobe suspicious for hepatocellular carcinoma (white arrows). CT: computed tomography

**Figure 9:**
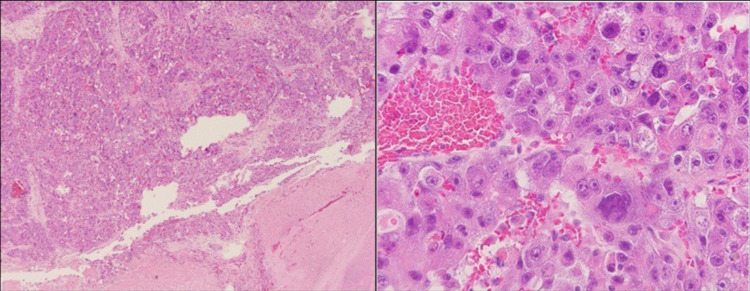
Inferior vena cava thrombus biopsy. Left image: low magnification of neoplastic hepatocytes adjacent to a blood clot. Right image: high magnification of neoplastic hepatocytes with enlarged, irregular nuclei and prominent nucleoli.

**Figure 10:**
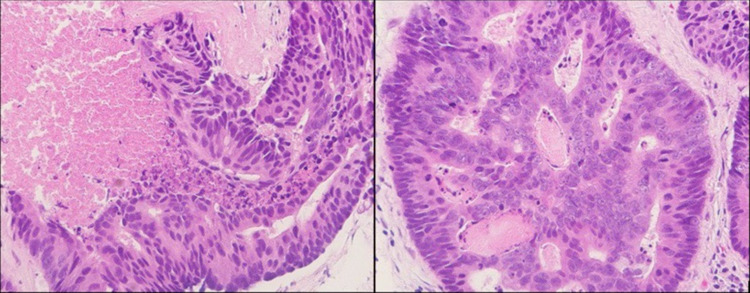
Colon biopsy. Left and right images show high magnification of neoplastic colonic epithelium with variation in nuclear size and open chromatin, as well as adjacent necrosis commonly seen with colorectal adenocarcinoma.

A 67-year-old African American male initially presented with bilateral leg swelling. A CT angiogram of the abdomen showed a lesion in the right liver lobe suspicious for HCC, as well as a clot with extension into the inferior vena cava (IVC) (Figure 8). The patient underwent angiovac evacuation of the thrombus and the specimen was sent for pathology, which showed high-grade carcinoma (Figure 9). The diagnosis of HCC was confirmed, and the staging was 2B. He was subsequently started on lenvatinib with good tolerance. He also received multiple local radiation treatments targeted at the tumor and the tumor extension into the thrombus over a six-month period. His lower extremity swelling improved, and his functional status was equivalent to an Eastern Cooperative Oncology Group score of 2. He was evaluated for TARE therapy but was not deemed a good candidate for this therapy. A year later, the patient presented with rectal bleeding and subsequently underwent a colonoscopy that found a sessile, ulcerated, non-obstructing medium-sized mass in the recto-sigmoid that was biopsied. Biopsy showed colorectal adenocarcinoma with extensive tumor necrosis (Figure 10). CT of the abdomen done at this time showed that the hepatic tumor had not changed in size from diagnosis. AFP was measured and found to be rising from initial values (initially 1,416, decreased to 213 with treatment, and increased back up to 1,002 with recurrence). Given the lack of tumor regression in size and rising AFP values, the patient was switched over to immunotherapy and started on nivolumab, which he continues to take. Following the completion of radiation therapy, the patient will be evaluated for further surgical intervention.

Case C

**Figure 11:**
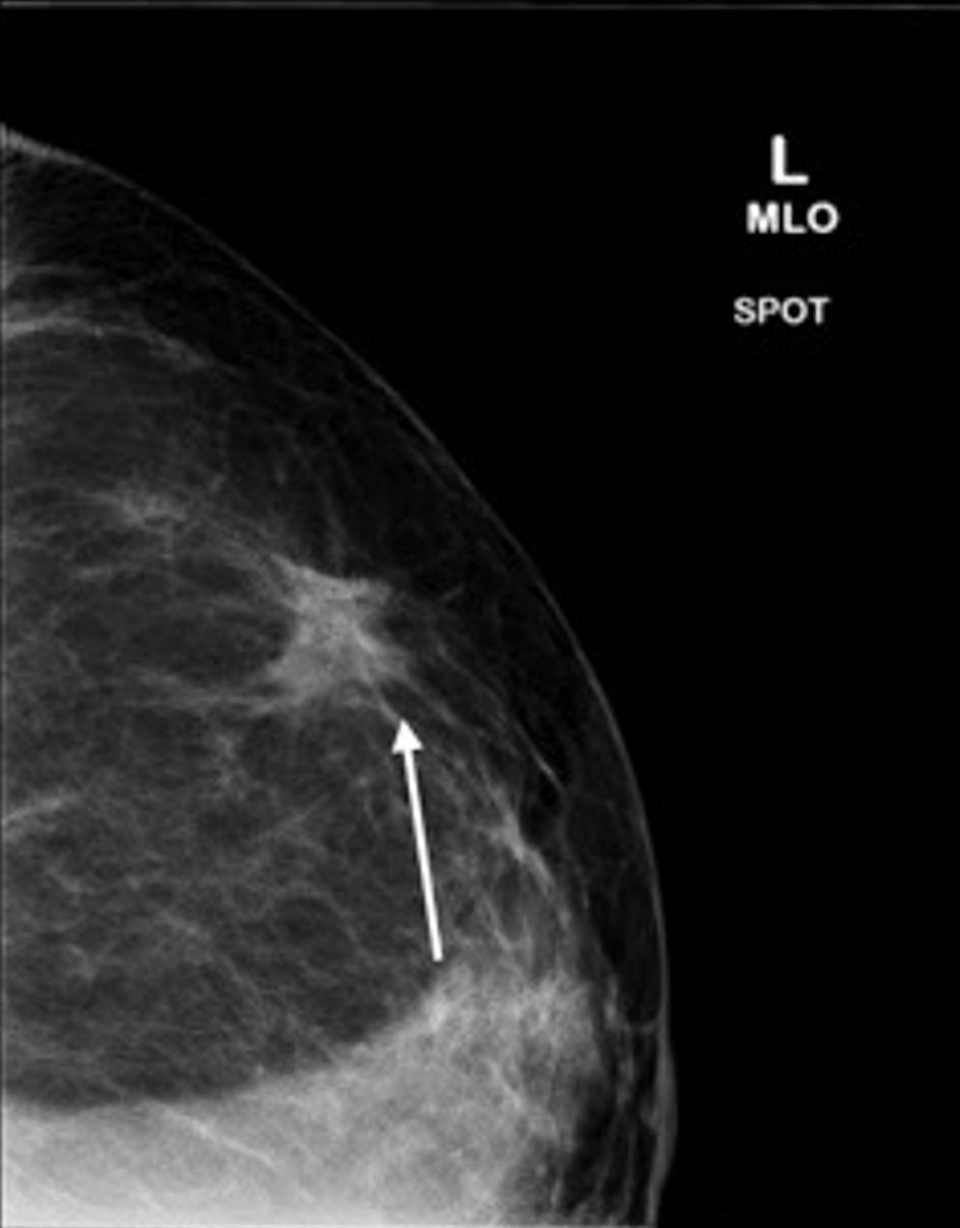
Mammogram showing the left breast spiculated mass ​measuring 1.4 × 1.4 × 1.3 cm in the left outer region (white arrow).

**Figure 12:**
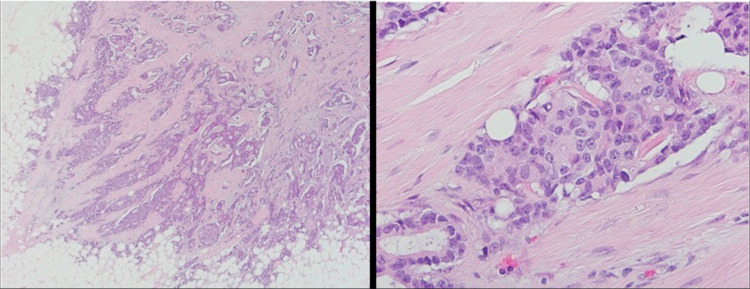
Ultrasound-guided biopsy of the left breast. Left image: low magnification showing invasive/neoplastic ductal cells, with surrounding fibrosis, going into adjacent fat. Right image: high magnification of neoplastic cells with nuclear changes, such as membrane irregularity and prominent nucleoli.

**Figure 13:**
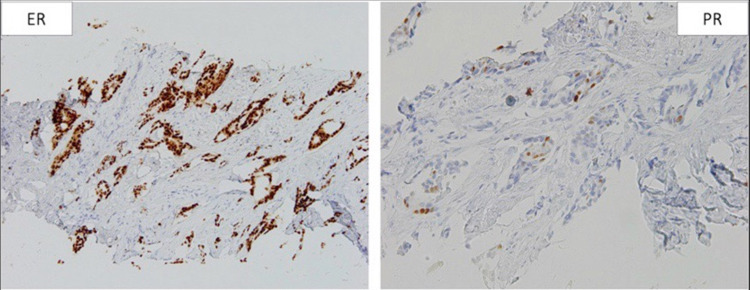
ER/PR staining of tissue from the initial breast biopsy. Left image: positive ER staining in 100% of cells. Right image: positive PR staining in 2% of cells. ER: estrogen receptor; PR: progesterone receptor

**Figure 14:**
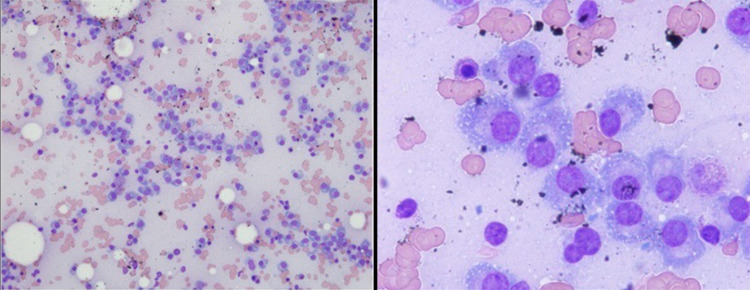
Bone marrow biopsy. Left image: low magnification of bone marrow aspirate showing abundant plasma cells. Right image: high magnification showing characteristic features of plasma cells with eccentrically located nuclei and perinuclear hofs.

**Figure 15:**
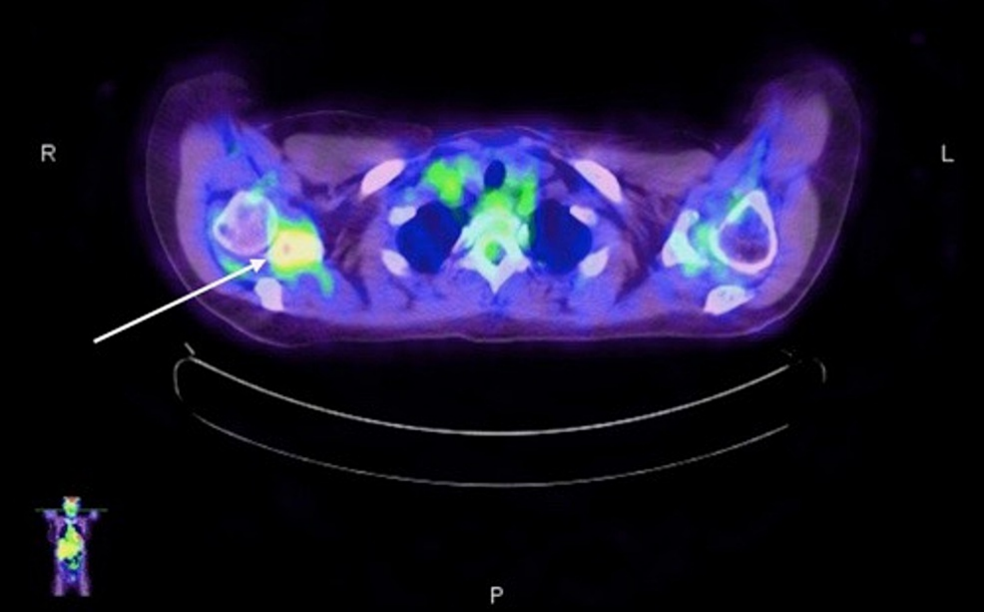
PET scan showing a hypermetabolic area in the right axillary lymph node (white arrow). PET: positron emission tomography

**Figure 16:**
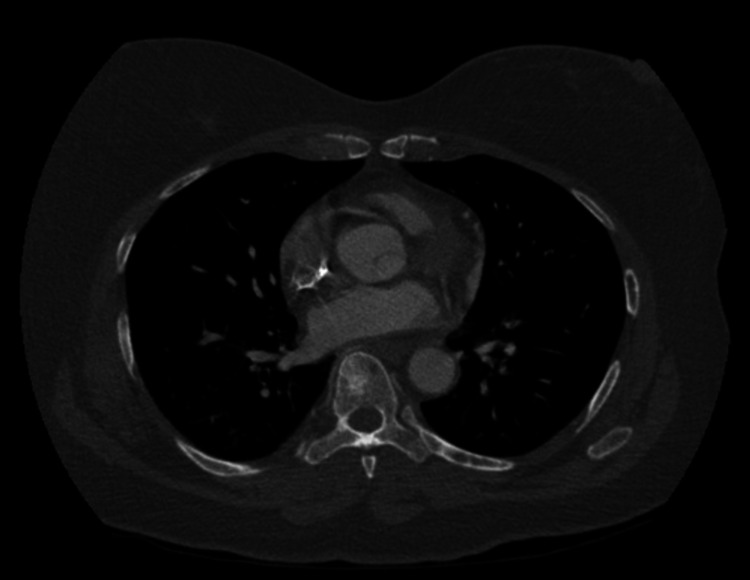
CT angiogram of the chest showing diffuse sclerotic bone lesions consistent with metastatic disease. CT: computed tomography

**Figure 17:**
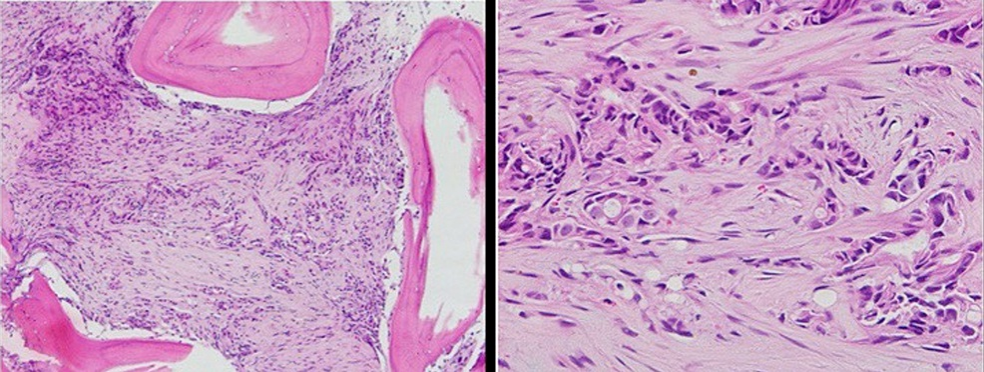
Right posterior iliac bone biopsy. Left image: low magnification showing significant marrow fibrosis with associated neoplastic cells. Right image: high magnification showing neoplastic cells with some tubule formation.

**Figure 18:**
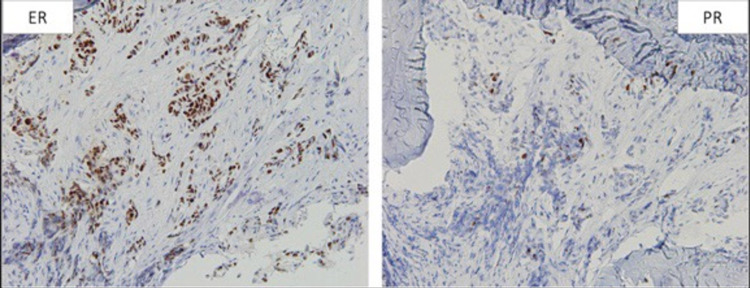
ER/PR staining of the tissue from bone biopsy, which was found to be similar to the initial staining. Left image: positive ER staining in 80% of cells. Right image: positive PR staining in 5% of cells. ER: estrogen receptor; PR: progesterone receptor

**Figure 19:**
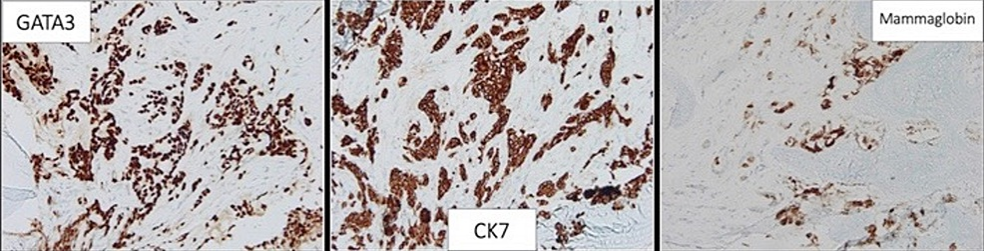
Right posterior iliac bone biopsy. Positive transcription factor GATA3, CK7, and mammaglobin staining, consistent with breast primary origin. CK: cytokeratin

A 60-year-old African American female underwent a screening mammogram that showed a left breast spiculated mass ​measuring 1.4 × 1.4 × 1.3 cm in the left outer region (Figure 11). She underwent an ultrasound-guided biopsy that confirmed the diagnosis of invasive ductal carcinoma of the left breast (Figure 12). The mass was estrogen receptor (ER) and progesterone receptor (PR) positive and human epidermal growth factor receptor 2 (HER 2) negative (Figure 13). She subsequently had a lumpectomy but declined to have radiation therapy. Approximately two years later she had a biopsy-proven local recurrence of an ER/PR-positive mass in the left breast and underwent another lumpectomy and sentinel lymph node biopsy which showed one positive node. The patient was referred back to radiation therapy for adjuvant radiation following breast-conserving surgery. She underwent approximately four weeks of radiation treatment but prematurely stopped after receiving only about one-third of her planned treatment due to intolerable side effects, including chest pain and anxiety attacks. Regarding hormone treatment, the patient tried tamoxifen, anastrozole, letrozole, and exemestane but all were discontinued due to intolerable symptoms such as rash, myalgia, and hair thinning. She further developed a benign breast mass on the opposing right side, and an MRI of the breast showed a stable mass on the right (BIRADS-2), findings consistent with a postoperative seroma. This was monitored with routine follow-up mammograms. The patient underwent an anemia workup that revealed borderline iron deficiency anemia as well as monoclonal gammopathy, immunoglobulin G lambda in both serum and urine with an M protein of 6.6 g/dL. The patient underwent a bone marrow biopsy that showed hypercellular marrow with marked infiltration by plasma cells (80%) (Figure 14). Flow cytometry confirmed monoclonality with a sample showing cluster of differentiation (CD) 38+, CD56+, and negative for CD19/20. The patient subsequently underwent a PET scan showing a hypermetabolic right axillary lymph node and diffuse osseous metastasis (Figure 15). A right axillary lymph node biopsy was done and was negative for malignancy. She began treatment for multiple myeloma with denosumab resulting in improvement in her hemoglobin, creatinine, and calcium levels. She tolerated treatment well completing six cycles overall. She was later transitioned to lenalidomide. She was evaluated for a bone marrow transplant but ultimately declined to proceed due to a lack of social support. Repeat bone marrow biopsy showed a normocellular marrow with a normal karyotype. The following year, the patient presented to an emergency room with a chief complaint of dyspnea and generalized body pain. A CT angiogram of the chest, abdomen, and pelvis showed clear lungs with no pulmonary embolism but scattered sclerotic bone lesions consistent with metastatic disease (Figure 16). She underwent a right posterior iliac bone biopsy that showed metastatic adenocarcinoma consistent with breast origin: ER/PR+, HER2-, positive transcription factor GATA3, cytokeratin (CK) 7, and mammaglobin staining (Figures 17, 18, 19). Based on the bone marrow findings of a higher disease burden for breast cancer, multiple myeloma treatment was held, and the patient was started on abemaciclib with exemestane added to reduce adverse effects. The patient continues treatment with abemaciclib and continues to follow up regularly with her oncologist.

## Discussion

MPMN was first described by Billroth in 1889 but the diagnostic criteria were not established until 1932 by Warren and Gates [1,5]. The overall incidence of MPMN is known to be up to 17% based on the site of primary malignancy [6], and the incidence is expected to rise as cancer screening and therapeutics continue to improve leading to longer survival.

While health disparities in single primary cancers are well established, these disparities are less studied in multiple primary malignancies. Regarding the overall incidence of multiple primaries, black patients have a lower incidence compared to white patients [6]. Several factors may play a role in the lower incidence of MPMN in blacks, one being differences in access to care resulting in underdiagnosed MPMN. Black patients also have lower relative survival in MPMN [6], similar to trends in single primary malignancy [2].

In a large cohort of African Americans and white patients with multiple myeloma, the incidence of second primary malignancy was studied. This study found that while the overall incidence of second primary malignancy was not higher in African Americans, the incidence of prostate cancer was significantly higher when compared to white males [3]. This may signal that while overall incidence may be lower in African Americans for MPMN, when the data is broken down by gender and the specific type of cancer, there may be additionally identified racial disparities.

Our first case (Case A, previously published [4]) describes a patient with non-small-cell adenocarcinoma who developed metachronous HCC. The management of MPMN presents various challenges to clinicians due to the lack of developed guidelines to help steer management, but NGS played an important role in this case. Based on an identified EGFR mutation, our patient was initially treated with gefitinib, an epidermal growth factor receptor tyrosine kinase inhibitor. Following the diagnosis and sequencing of a secondary HCC, a new gatekeeper mutation of the T790M missense variant was identified leading to a switch in agents from gefitinib to osimertinib. Osimertinib has shown to have greater efficacy in patients with T790M-positive advanced non-small-cell lung cancer [7]. Overall, this provides a good example of tumor agnostic therapy, where treatment is based on the cancer’s molecular factors as opposed to tumor location.

The second case (Case B) presented a patient who developed primary HCC and then a year later developed metachronous colorectal adenocarcinoma. The risk of developing a second primary cancer varies between 1% and 16% based on the site of primary cancer [6]. Primary HCC has the lowest risk for developing a second primary cancer at 1%, highlighting the rare occurrence seen in our patient. Based on the patient’s inoperable HCC, he was started on first-line treatment with lenvatinib. The US Food and Drug Administration approved nivolumab for the treatment of HCC that failed to respond to vascular endothelial growth factor antagonists such as sorafenib or lenvatinib [8].

Our third case (Case C) highlights an African American female diagnosed with invasive ductal carcinoma of the left breast initially treated with surgery and followed by adjuvant radiation therapy. The patient went on to develop multiple myeloma, and due to a bone marrow biopsy showing a high disease burden of breast cancer, multiple myeloma treatment was held and the patient was started on abemaciclib. Abemaciclib is indicated in hormone receptor-positive and HER2-negative patients with advanced breast cancer, and it has been shown in Monarch 1,2,3 to increase overall survival when administered with an aromatase inhibitor [9]. In rare cases of MPMN, a targeted approach that quantifies which malignancy has a higher tumor burden and prioritizes treatment can provide a framework for clinicians in guiding management. Of note, this patient’s lack of social support impacted her ability to complete all indicated treatments such as the bone marrow transplant. Social factors play an important role in the management of cancers and can play a role in developing multiple primary malignancies such as in this patient. Underserved populations are at greater risk of having their cancer treatment being affected by social factors, and special attention should be paid to this by clinicians.

## Conclusions

While the incidence of MPMNs is widely available, the incidence rate within African Americans and other underserved groups is not well studied. This case series features three African Americans with MPMNs that are rarely described in the literature and outlines the management of these challenging clinical scenarios. Further research is required to explore if there is increased risk within blacks and establish the race-specific risk factors associated with MPMN.
